# Camphor—A Fumigant during the Black Death and a Coveted Fragrant Wood in Ancient Egypt and Babylon—A Review

**DOI:** 10.3390/molecules18055434

**Published:** 2013-05-10

**Authors:** Weiyang Chen, Ilze Vermaak, Alvaro Viljoen

**Affiliations:** Department of Pharmaceutical Sciences, Faculty of Science, Tshwane University of Technology, Private Bag X680, Pretoria 0001, South Africa; E-Mails: chenw@tut.ac.za (W.C.); vermaaki@tut.ac.za (I.V.)

**Keywords:** camphor, *Cinnamomum camphora*, biological activity, synthesis, toxicity

## Abstract

The fragrant camphor tree (*Cinnamomum camphora*) and its products, such as camphor oil, have been coveted since ancient times. Having a rich history of traditional use, it was particularly used as a fumigant during the era of the Black Death and considered as a valuable ingredient in both perfume and embalming fluid. Camphor has been widely used as a fragrance in cosmetics, as a food flavourant, as a common ingredient in household cleaners, as well as in topically applied analgesics and rubefacients for the treatment of minor muscle aches and pains. Camphor, traditionally obtained through the distillation of the wood of the camphor tree, is a major essential oil component of many aromatic plant species, as it is biosynthetically synthesised; it can also be chemically synthesised using mainly turpentine as a starting material. Camphor exhibits a number of biological properties such as insecticidal, antimicrobial, antiviral, anticoccidial, anti-nociceptive, anticancer and antitussive activities, in addition to its use as a skin penetration enhancer. However, camphor is a very toxic substance and numerous cases of camphor poisoning have been documented. This review briefly summarises the uses and synthesis of camphor and discusses the biological properties and toxicity of this valuable molecule.

## 1. Introduction

“Smell is a potent wizard that transports you across thousands of miles and all the years you have lived. The odors of fruits waft me to my southern home, to my childhood frolics in the peach orchard. Other odors, instantaneous and fleeting, cause my heart to dilate joyously or contract with remembered grief. Even as I think of smells, my nose is full of scents that start awake sweet memories of summers gone and ripening fields far away.” The human sense of smell has a powerful effect on our basic emotions, as eloquently described by Helen Keller [1]. Vernet-Maury *et al*. [2] tried a new approach to prove this phenomenon by analysing autonomic nervous system (ANS) responses, whilst study participants completed a hedonic scale to rate the “pleasantness” or “unpleasantness” of odours. The pooled results revealed a high correlation between the hedonic evaluation and basic emotions. Camphor was an intermediate, associated with “happiness” or “surprise”—characteristics of the “pleasant” odourants (ethyl acetoacetate and lavender), but also linked to “sadness”, which differentiated it from these odours. Despite the “sadness” rating, the emotions associated with camphor were all different from those associated with acetic and butyric acids, used as the “unpleasant” odourants [2]. Whether camphor makes one think of mothballs or your grandmother, it must surely be one of the most distinctive odours, even having its own adjective to describe it, *i.e.*, camphoraceous.

**Figure 1:**
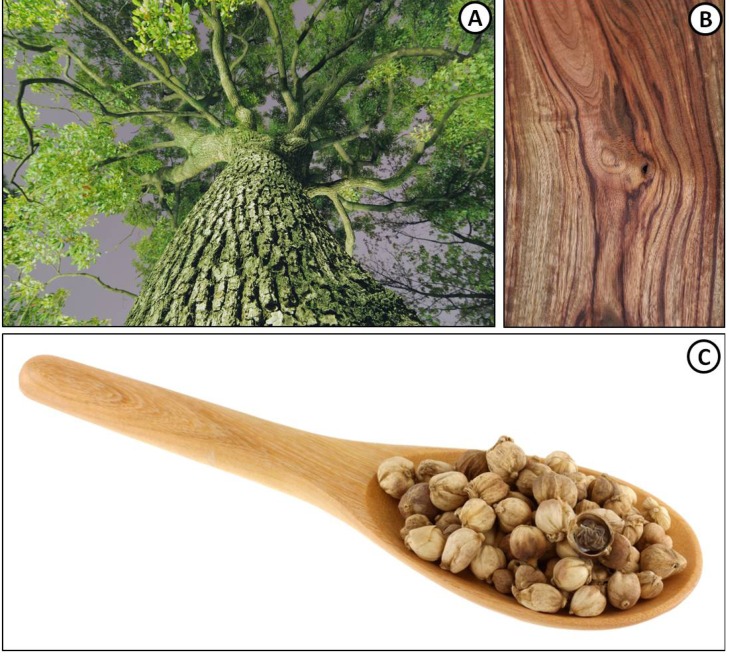
*Cinnamomum camphora* tree, wood and seeds.

The fragrant camphor tree, *Cinnamomum camphora* (L.) J. Presl (Lauraceae), occurs naturally in Asian countries including Japan, Taiwan and China, but has been naturalised in other parts of the World. The tree is large with pale brown bark, dark green to yellowish leaves (Figure 1A) and small white flowers followed by small purple berries. All the plant parts have the distinctive, easy-to-recognise camphoraceous odour. The essential oil is distilled from the wood (Figure 1B) which yields the active ingredient (1*R*)-(+)-camphor, *i.e.*, natural camphor [3,4].

Camphor has a long-valued history for its extensive and diverse uses in the East: the Chinese used camphor as a circulatory stimulant and analeptic, whilst the Japanese used it in torch-light material and added small quantities to fireworks to make them brighter [5]. Camphor was used as a fumigant during the Black Death, a plague that spread through Europe in the 14th century, as well as during outbreaks of smallpox and cholera. Rosewater together with camphor as a perfume ingredient was sprinkled over corpses before shrouding [6]. In India, camphor is commonly burnt in temples during religious rituals because unlike any other aromatic smoke, camphoric fumes are non-irritant to eyes [7]. Camphor has been widely used as a fragrance in cosmetics, as a flavouring food additive and as a preservative in confectionary goods; in homes it is commonly used as an insect repellent, a plasticiser and as an intermediate in the synthesis of aroma chemicals [7,8]. Camphor is one of the most well-known and widespread commercially important aroma chemicals, with an annual market value of 80–100 million US$ [9].

Camphor exhibits several biological properties such as antimicrobial, antiviral and antitussive effects [10,11,12,13,14]. Camphor is a common ingredient in modern medicine in topically applied analgesics and rubefacients for treatment of minor muscle aches and pains and it is reported that camphor has been used to relieve pain caused by breast engorgement by intramuscular injections [15]. It has been applied as a topical anti-infective and anti-pruritic and internally as a stimulant and carminative [3]. However, camphor is poisonous when ingested and can cause seizures, confusion, irritability and neuromuscular hyperactivity. The lethal dose in humans is reported to be 50–500 mg per kg bodyweight [16]. This review unites the physical properties, chemistry and synthesis of camphor and focuses on the research findings detailing its numerous biological properties as well as the toxicity profile.

## 2. Physical Properties and Sources of Camphor

**Figure 2:**
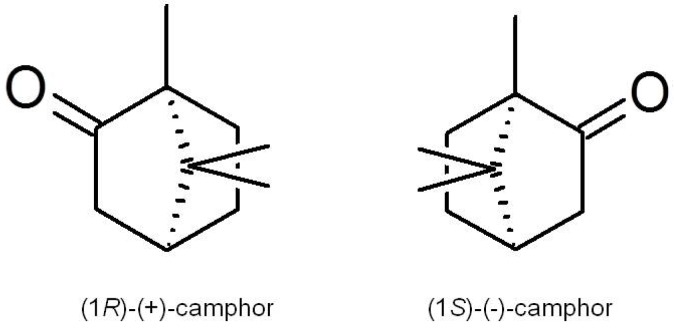
The chemical structure of the (1*R*)-(+) and (1*S*)-(−) enantiomers of camphor.

Camphor is a waxy, white or transparent solid with a strong aromatic odour [17] which sublimates at room temperature and melts at 180 °C [7]. It is practically insoluble in water, but soluble in alcohol, ether, chloroform and other organic solvents. It is a terpenoid (1,7,7-trimethylbicyclo[2.2.1]-2-heptanone) with a chemical formula of C_10_H_16_O and exists in two enantiomeric forms: (1*S*)-(−)-and (1*R*)-(+)-camphor (Figure 2). These two enantiomers have a similar camphoraceous odour, but how the stereochemistry impacts on the biological activity is still unknown [18,19]. Synthetic camphor is synthesised mainly from α-pinene obtained from turpentine oil, whilst natural camphor, *i.e.*, (+)-camphor, is obtained through distillation of the wood from the camphor laurel tree (*Cinnamomum camphora*) found especially in Borneo and Taiwan; the Borneo camphor tree (*Dryobalanops aromatica*) and the East African camphorwood tree (*Ocotea usambarensis*). In Asia, a major source of camphor is camphor basil (*Ocimum kilimandscharicum*) [20]. Camphor is also present as a major essential oil component of many aromatic plant species [10,11,12,13,14,21,22,23,24].

## 3. Biosynthesis and Chemical Synthesis of Camphor

**Scheme 1:**
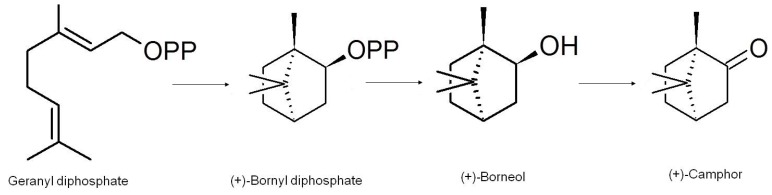
The biosynthesis of camphor (adapted from [28]).

The biosynthesis of camphor was extensively investigated and elucidated by Croteau *et al*. [25,26,27] in their work on *Salvia officinalis* [25,26,27]. After several elegant experiments they determined the basic biosynthesis of camphor (Scheme 1) and the enzymes involved to be as follows: Camphor is biosynthetically produced in plants by way of the biotransformation of the starting material geranyl diphosphate (GPP) which is the preferred substrate. Cyclisation of geranyl diphosphate, by the enzyme (+)-bornyl diphosphate synthase yields (+)-bornyl diphosphate. (+)-Bornyl diphosphate is then hydrolysed to (+)-borneol through the action of bornyl-diphosphate diphosphatase. The last step is catalysed by (+)-borneol dehydrogenase as it oxidises (+)-borneol to (+)-camphor [25,26,27].

**Scheme 2:**
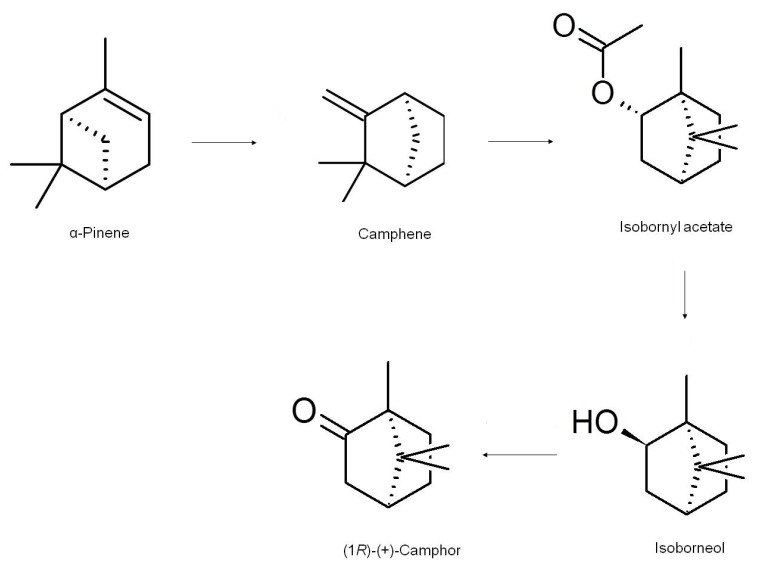
The chemical synthesis of camphor.

The synthetic production of camphor involves using turpentine oil as a starting material. Turpentine is used as the source of α-pinene through a distillation process; α-pinene is converted into camphene through the catalysis of a strong acid with acetic acid as the solvent; the camphene then undergoes Wagner-Meerwein rearrangement into the isobornyl cation, which is captured by acetate; the isobornyl acetate subsequently formed is hydrolysed to isoborneol, which is finally converted to camphor through dehydrogenation [20] (Scheme 2). The synthetic route from α-pinene produces a racemic mixture, *i.e.*, a 1:1 ratio of (−) and (+)-camphor.

## 4. Camphor as a Chiral Starting Material and Auxiliary

Monoterpenes are ideal for use as chiral synthons, or building blocks, for synthesis of other compounds due to their low cost and abundant availability. Camphor and camphor-derived analogues are used in a myriad of chemical reactions, as chiral auxiliaries in asymmetric synthesis or as catalysts. There are numerous reports on the use of camphor imine as a template to direct enantioselective alkylation for the synthesis of α-amino acids, α-amino phosphonic acids, α-substituted benzylamines and α-amino alcohols. Camphenesulfonic acid, a camphor-derived organic acid, is useful for chiral reductions such as the resolution of racemic bases [9]. Other camphor analogues, with the C-8 methyl group replaced by larger and more sterically bulky groups, can significantly increase the stereoselectivity through alkylation resulting in much higher enantiomeric purity of the products [29]. Camphor can be used as the starting material for the synthesis of aromatic steroids. From the readily available (1*S*)-(−)-camphor, direct functionalisation at the C-9 methyl group was accomplished through three efficient steps to give (−)-π-bromocamphor with complete retention of chirality. The bromo-ketone was converted to the cyanketone by a number of transformations. The ketone followed by POCl_3_-pyridine treatment to give the tricyclenone was then reacted with methyl vinyl ketone, a critical C, D intermediate in aromatic steroid synthesis. At this stage, all the chiral centres in the final product are correctly configured, the tricyclo-ring system is combined with an intermediate in aromatic steroid synthesis and all the chiral centres have the appropriate functionalities to allow completion of the steroid synthesis [30]. Vitcamphor, discovered in the urine of patients taking camphor and then extracted from dogs consuming camphor, has been used in acute heart failure as a cardiotonic drug; however, side-effects were reported as the purity of this product was low. Shuang *et al*. [31] chemically synthesised vitcamphor from camphor through bromination, followed by esterification, reduction, hydrolysis and oxidation resulting in a molecule with a purity of greater than 96% and an increased yield of 6% [31]. Camphor and its analogues are versatile molecules which can be used as either templates or starting materials in the synthesis of new molecules, or as catalysts in various chemical reactions.

## 5. The Biological Properties of Camphor

### 5.1. Antimicrobial Activity

#### 5.1.1. Antibacterial and Antifungal Activities

Plants have been a valuable source of natural products for maintaining human health and the use of plant compounds for their antimicrobial activity has gradually increased worldwide. Numerous investigations have shown various essential oils of several species containing camphor as the major components, exhibited antimicrobial activity [14,32,33,34,35]. The composition of essential oil from the aerial parts of sweet wormwood (*Artemisia annua*) includes camphor (44%), germacrene D (16%), *trans*-pinocarveol (11%), β-selinene (9%), β-caryophyllene (9%) and artemisia ketone (3%). Significant activity of the essential oil was noted against the Gram-positive bacteria, *Enterococcus hirae*, as well as against the fungi *Candida albicans* and *Saccharomyces cerevisiae* using the liquid diffusion method [10].

Some studies found camphor as a single compound only exhibited weak antimicrobial activity [36,37]. Greek sage (*Salvia fruticosa*) essential oil, containing camphor as the main component, exhibited poor activity against all of the bacteria tested (*Escherichia coli*, *P. aeruginosa*, *Salmonella typhimurium*, *Staphylococcus aureus*, *Rhizobium leguminosarum* and *Bacillus subtilis*) [38]. Santoyo *et al*. [39] investigated the antimicrobial activity of rosemary essential oil obtained via supercritical fluid extraction and molecules including camphor against *S. aureus*, *B. subtilis*, *E. coli*, *P. aeruginosa*, *C. albicans* and *Aspergillus niger* by the disc diffusion and broth dilution methods. The test samples were active against all the test organisms with the most susceptible organism being *S. aureus* and the least susceptible *A. niger*. The standards exhibit activity against all the micro-organisms tested in the order of effectiveness: borneol > camphor > verbenone. In another study, a yarrow variety (*Achillea sintenisii*) was found to contain camphor (14.8%) as its major component. The essential oil was further fractionated and the antibacterial and antifungal activities determined against a variety of micro-organisms. Data analysis revealed camphor to be the more active compound together with 1,8-cineole, as notable activity against *C. albicans* and *C. krusei* was reported. The fractions showed the same or higher activity than the neat essential oil in the majority of cases [40]. Mevy *et al*. [41] confirmed that elemol, 1,8-cineole, camphor and *p*-cymene can be considered as the principal antimicrobial components of tea bush (*Lippia chevalieri*) oil. The antimicrobial activity of rosemary (*Rosmarinus officinalis*) and several other oils against organisms implicated in meat spoilage was investigated by Ouattara *et al*. [42]. In a 1/100 dilution, rosemary (*Rosmarinus officinalis*) oil, containing mainly camphor, was one of the most efficient antibacterial oils having antibacterial activity against two Gram-negative (*Pseudomonas fluorescens* and *Serratia liquefaciens*) and four Gram-positive (*Brochothrix thermosphacta*, *Carnobacterium piscicola*, *Lactobacillus curvatus*, and *Lactobacillus sake*) bacteria [42]. The essential oils of *Salvia macrochlamys* and decorative sage (*S. recognita*), rich in camphor (11% and 42%, respectively), exhibited no antimicrobial activity against methicillin-resistant *S. aureus*, *Mycobacterium intracellulare*, *Cryptococcus neoformans* and *Aspergillus fumigatus,* but moderate antifungal activity against *Colletotrichum acutatum*, *C. fragariae*, and *C. gloeosporoides* was noted at a concentration of 200 µg/mL. However, in the same study, (−)-camphor and (+)-camphor tested singularly exhibited no activity against the test bacteria and fungi [43]. Viljoen *et al*. [13] determined, using time-kill studies, that a synergistic antimicrobial effect occurs between 1,8-cineole and (−)-camphor. These two compounds are the two major essential oil components of the mountain daisy (*Osmitopsis asteriscoides*). The study showed that both (+)-camphor and (−)-camphor have negligible antifungal activity on *C. albicans*, whereas in the case of (−)-camphor combined with 1,8-cineole, a total reduction of colony forming units (CFUs) was observed at 15 min. It was deduced therefore that camphor may act in a synergistic manner with other essential oil components possessing antimicrobial activity [13].

#### 5.1.2. Antiviral Activity

Viral diseases are an increasing health concern worldwide and there has been an intensive search for more effective but less toxic antivirals than those currently used. Aromatic plants, especially their essential oils, are known to exhibit antiviral properties. Sivropoulou *et al*. [38] investigated the antimicrobial, cytotoxic and antiviral activities of Greek sage (*Salvia fruticosa*) essential oil. The results demonstrated the essential oil of Greek sage (*S. fruticosa*) and its four main components (1,8-cineole, α- and β-thujone, and camphor) exhibited high levels of virucidal activity against herpes simplex virus-1, however, this positive effect was accompanied by cytotoxic activity against African Green Monkey kidney (Vero) cells. Lavender cotton (*Santolina insularis*) essential oil, which is rich in camphor, deactivated herpes simplex type-1 (HSV-1) and type-2 (HSV-2) *in vitro* using plaque reduction assays with an IC_50_ value of 0.88 µg/mL for HSV-1 and 0.7 µg/mL for HSV-2. Reduction of plaque formation assays showed inhibition of cell-to-cell transmission of both HSV-1 and HSV-2 [44].

### 5.2. Antitussive Activity

Coughing is a very common clinical symptom with largely ineffective current therapies. Aromatic vapours have been widely used in the symptomatic treatment of upper respiratory tract infections due to their known antitussive effects. Burrow and co-workers [45] investigated the effects of camphor vapour on nasal resistance to airflow and nasal sensation of airflow. Inhalation of camphor had no effect on nasal resistance to airflow, but a cold sensation in the nose with the sensation of improved airflow was described. The results indicated that camphor stimulated cold receptors in the nose [45]. Laude *et al*. [46] reported the action of camphor on the cough reflex in conscious guinea-pigs. Three concentrations (50, 133 and 500 mg/L) of camphor vapour were administered and 500 mg/L camphor significantly reduced (33%) cough frequency. An increase in cough latency coincided with the reduction in cough frequency. Further studies revealed camphor activated cold receptors now identified as TRPM8, the minty-cool ion channel, but the mechanism whereby TRPM8 activation inhibits cough is still not understood [47,48]. In another study, camphor was used to synthesise camphor lactam (α-camphidone) by treatment with hydroxylamine-*O*-sulfonic acid and glacial acetic acid with a Beckmann-like rearrangement in structure. Both camphor and camphor lactam were tested for their antitussive activity in guinea-pigs with citric-acid induced cough. It was noted that this minor modification in chemical structure significantly increased cough latency whilst reducing cough frequency. In addition, prior exposure to the camphor lactam at concentrations of 125, 250, and 500 μg/L had a higher inhibitory cough response compared to camphor [49].

### 5.3. Anti-Nociceptive Activity

Camphor has an extensive history in its use as a topical analgesic in balms and liniments. In 1990, Green [50] found camphor to be a relatively weak sensory irritant having a modest excitatory effect on thermosensitive (and perhaps nociceptive) cutaneous fibers. Xu *et al*. [51] further investigated the mechanism of camphor anti-nociceptive activity and reported that camphor activated and de-sensitised the capsaicin receptor (TRPV1) whilst inhibiting the garlic receptor (TRPA1). Both are part of the recently elucidated transient receptor potential (TRP) superfamily, a group of structurally similar ion channels, heavily expressed in nociceptive sensory neurons. Therefore, it is possible that the analgesic effects of camphor may be due to de-sensitisation of TRPV1 and blocking of TRPA1 [51]. The pain-relieving effects of California sagebrush (*Artemisia californica*), containing the two major compounds 1,8-cineole (24%) and camphor (18%), were reviewed. Anecdotal use reported successful pain relief in all cases for patients suffering from lower back pain, arthritis, bruises, muscle and ligaments strains, broken bones and even cancer. An alcoholic liniment provided rapid pain relief lasting 2–3 h with an onset of action of 20 min. The anti-nociceptive activity of terpenoids is as a result of binding to TRPV1, TRPV3 and TRPM8 receptors. Camphor is a known agonist of TRPV2, TRPA1 as well as TRPV1 quickly deactivating TRP channels resulting in long-term pain relief [52].

### 5.4. Antimutagenic and Anticancer Activity

Few animal studies demonstrating the potential of camphor in the treatment of cancer have been conducted, but those undertaken included improvement of immune function [53], enhancement of enzymatic breakdown of carcinogens [54] and the increased susceptibility of cancer cells to radiation [55]. Goel *et al*. [56] demonstrated that camphor had a radiomodifying effect. An increase in the frequency of sister-chromatid exchanges (SCE) in mice bone marrow cells occurs after exposure to gamma radiation, but after a single dose of camphor, administered at 0.5 µM/g bodyweight, this frequency was significantly low. Kanematsu and Shibata [57] reported on possible DNA damage as shown by a positive result of the rec-assay using two strains of *Bacillus subtilis*. Camphor, often used in endodontic formulations, presented a positive result in the “rec-assay”, showing that camphor may cause genetic toxicity in cells, however, drugs showing positive results do not necessarily cause tumour formation. This shows that more studies on the genotoxicity of camphor are required and that camphor should be used with care. Cultivated sage (*Salvia officinalis*) rich in camphor reduced UV-induced mutagenesis when screened with the repair-proficient strain, and had no effect on spontaneous mutation frequency in mismatch repair-deficient strains. It also enhanced the formation of Lac^+^ recombinants, but not as a consequence of SOS induction. This result suggested a protective effect through enhanced re-combinational repair [58]. In a subsequent study, Vuković-Gacić *et al*. [59] investigated the inhibitory potential of cultivated sage essential oil and its monoterpenes on UV-induced mutations tested with SY252 and D7. Camphor showed antimutagenic effects at very low concentrations compared with other monoterpenes screened (about 40% reduction of UV-induced revertant at 0.5 and 1 μg/plate), although higher concentrations failed to increase antimutagenic effects. Nikolić *et al.* [60] demonstrated that camphor can reduce UV/4NQO mutagenesis in the NER+, but not the NER− strain of *Escherichia coli* and increased spontaneous and UV-induced recombination in recA730 and recA+ cells. Low doses of camphor are antigenotoxic against 4NQO in mammalian cells and stimulate DNA repair, acting as a bioantimutagen. De-Oliveira *et al*. [61] hypothesised based on previous findings how the genotoxicity of mutagens may be modulated through cytochrome P4502B subfamily enzyme inhibition. In a study including various monoterpenes using pentoxyresorufin-*O*-depenthylase (PROD) as a model substrate for cytochrome P4502B1-enzymes, camphor was found to have an inhibitory effect on the PROD enzyme with an IC_50_ value of 7.89 μM. Through this mechanism of action it is possible for camphor to be considered antimutagenic [61], but more studies are required.

### 5.5. Insecticidal Activity

Certain currently used synthetic pesticides threaten the integrity of the earth’s ozone layer and other environmental buffers, therefore alternatives to these commercial chemicals are urgently needed [62]. Essential oils are considered good candidates because of their low toxicity to mammals, high volatility, ready availability in tropical countries and economical viability [63,64]. Monoterpenoids believed to aid plants in chemical defence against phytophagous insects are capable of toxic interference with the biochemical and physiological functions of herbivorous insects [65,66]. Numerous studies have indicated that camphor has insect repellent activity against stored-product pests. Using contact toxicity, grain treatment and repellency assays, the essential oil of camphor basil (*Ocimum kilimandscharicum*) and its major component, camphor, were investigated against four beetles. The doses of 100 mg/filter paper and 100 µg/insect induced over 93% and 100% mortalities in *Sitophilus granarius*, *S. zeamais* and *Prostephanus truncatus*, but only 70% and 100% mortality in *Tribolium castaneum* after 24 h exposure. Development of eggs and immature stages within grain kernels, as well as progeny emergence, was completely inhibited in camphor-treated grain [67]. Bekele and Hassanali [68] revealed the insecticidal activity of camphor basil (*O. kilimandscharicum*) against *Rhyzopertha dominica* and *S. zeamais* was due to camphor and the combined effects of different components, but camphor had no effect on the rice weevil (*Sitophilus oryzae*) with an LC_50_ of greater than100 μL/L. Another report indicated that camphor, as a pure compound, showed contact and fumigant activity against *S. oryzae* and *Rhyzopertha dominica*, but had no effect on *Tribolium castaneum* after 24 hours exposure at a dose of 0.1 μL/720 mL volume [69]. Liska *et al.* [70] found camphor exhibited the highest mortality (78.5%) just after 24 h at the highest tested dose (10.0 μL/adult) for contact toxicity; for fumigant toxicity, camphor at its highest dose (120 μL/350 mL vol.) caused 93.5% mortality. These results were in agreement with an earlier report by Qiantai and Yongcheng [71], who observed that camphor, as the major isolate from essential oils of Chinese cinnamon (*Cinnamomum cassia*), Chinese star anise (*Illicium verum*) and camphor laurel (*Cinnamomum camphora*), showed contact efficacy against the lesser grain borer (*Rhyzopertha dominica*) and the maize weevil (*Sitophlus zeamais*). Insect repellent activity, rather than insecticidal activity, against the confused flour beetle (*Tribolium castaneum*) was noted. Exposure of the newly-laid eggs of the pulse beetle (*Callosobruchus chinensis*) to concentrations of 0, 12, 24, 48, and 96 ppm of camphor crystals in air-tight containers for one and four weeks resulted in 65.0% to zero (0%) hatching. Postembryonic development was affected when the newly-hatched larvae penetrated into the cowpea seeds; very few adults emerged from the infested seeds exposed to camphor [72].

Camphor was the most effective of the tested monoterpenoids to prevent the multi-coloured Asian lady beetle, *Harmonia axyridis* (Pallas), from overwintering in buildings as determined by the olfactometer bioassay [73]. It also exhibited fumigation toxicity against false powder post beetle (*Dinoderus bifloveatus*) after 48 hours exposure [74]. Several monoterpenes (e.g., 1,8-cineole, citronellol, α and β-pinene, linalool, isopinocamphone, camphor) tested against cattle-tick (*Boophilus microplus*) larvae and camphor, in a 60 min-period, was lethal to 100% of the larvae [75]. *Blattella germanica* (German cockroach) is one of the most important pests of the indoor environment, a major source of allergens and a potential carrier of faecal pathogens [76]. Using the filter-paper contact toxicity bioassay, both (1*R*)-(+)-camphor and (1*S*)-(−)-camphor were toxic against female *B. germanica* with LD_50_ values of 0.10 mg/cm^2^ and 0.13 mg/cm^2^, respectively. There was no significant difference in toxicity between (1*R*)-(+)-camphor and (1*S*)-(−)-camphor [77].

Mosquitoes are known disease vectors of malaria, haemorrhagic dengue and yellow fever, in addition to being considered a nuisance [78]. Most commercial mosquito repellents contain DEET (*N,N*-diethyl-*m*-methylbenzamide), but this compound exerts toxic reactions and may damage plastic, synthetic fabrics and painted surfaces [79]. Bioassays conducted on a number of essential oils showed repellency against mosquitoes attributed to the presence of its main compounds. However, it was noted that synergy between the essential oil components may lead to an increased repellant response; therefore, the neat oil may be more effective compared to the individual components [80]. A review published in 2011 noted 144 patent inventions containing plant essential oils for mosquito repellency. These patents, mostly from Asian countries, listed various essential oil components including amongst many others citronella (*Cymbopogon* spp.) and eucalyptus (*Eucalyptus* spp.), whilst camphor (*Cinnamomum camphora*) was mentioned in >10% of these patents [78]. As a major component of the essential oil of aromatic plants, camphor has shown repellency against *Anopheles culicifacies*, *Cx. quinquefasciatus* [81], *Anopheles gambiae* and *Anopheles funestus* [82,83]. It is evident that camphor has great potential for development as an alternative green commercial insect repellent to replace the harmful synthetic agents currently in use.

### 5.6. Cardiovascular Effects

For centuries, camphor has been used for the stimulation of heart and peripheral circulation. Osborne [84] reported that in cardiac failure and collapsed conditions characterised by cold skin, a feeble pulse and failing heart, the subcutaneous injection of camphor in sterile oil caused the surface of the skin to become flushed, dilated the peripheral blood vessels and improved the whole circulation. The results of controlled clinical studies on the cardiovascular effects of (+)-camphor have been published [85]. Belz and Loew [86] investigated the effects of (+)-camphor (extracted from fresh *Crataegus* berries) in orthostatic hypotension using independent, double-blind, randomised, placebo-controlled studies. It was determined that (+)-camphor, as well as the extract from fresh hawthorn (*Crataegus*) berries, contributed to the pressoric effects with (+)-camphor inducing the initial rapid effect and the extract is responsible for the long-lasting effect [86].

### 5.7. Camphor as a Potential Skin Penetration Enhancer

It has been suggested that terpenes, present in plant essential oils, are clinically acceptable skin penetration enhancers [87]. Previous studies also indicated menthol in combination with camphor enhanced the skin penetration of methyl salicylate and inhibited both the *in vivo* and *in vitro* hydrolysis of methyl salicylate to salicylic acid [88]. The efficacy of some terpenes on skin permeation of tea catechins and theophylline were systemically evaluated using a series of *in vitro* and *in vivo* methods [89]. It was found that all the evaluated terpenes had significant effects on the (+)-catechin delivery relative to the control. The rank order of enhancement was α-terpineol ≥ menthone >linalool > 1,8-cineole > farnesol ≥ fenchone > cymene ≥ nerolidol > (+)-limonene > camphor. Camphor and fenchone showed the least enhancement amongst the oxygen-containing monoterpenes, which may be related to their bicyclic structure.

Ramesh *et al*. [90] reported the flux of carvedilol obtained from solutions containing camphor, transcutol, *d*-limonene, carvone, labrasol and menthol were 7.81, 7.26, 6.52, 5.91, 4.21 and 2.28 times higher respectively, than that observed with the control, using excised rat abdominal skin mounted in Franz diffusion cells. Camphor showed maximum permeation and basil oil (*Ocimum basilicum*) containing methyl chavicol, eugenol, linalool, camphor and methyl cinnamate showed potential *in vitro* penetration enhancement of labetolol hydrochloride [91]. In a recent study, camphor was found to markedly prevent the permeation of benzocaine across the skin while promoting skin accumulation after 12 h [92].

### 5.8. Other Applications

Interestingly, according to Iranian folk medicine, camphor was used both as an aphrodisiac and antiaphrodisiac agent. The effect of camphor on the sexual activity of male rats was investigated by Jamshidzadeh *et al*. [93], by measuring the parameters mount latency and frequency as well as intromission latency and frequency. The results indicated enhanced sexual desire and performance when camphor was administered at 50 mg/kg. It was speculated that the effects of camphor may be due to modulation of the sympathetic nervous system, or its effect on serum testosterone levels [93]. Camphor could also have an effect on the reproductive function of the testes in mice, as it was revealed that administration of camphor to young male mice may result in vascularisation and proliferation of sexual cells which can affect maturation of seminiferous tubules [94].

Sweet wormwood (*Artemisia annua*) leaves and chemical constituents, including camphor, were investigated for its activity against coccidian parasites. A 5% dried leaf supplement addition to chick feed for 3 weeks resulted in infection protection against *Eimeria tenella* but not *Eimeria acervulina* or *Eimeria maxima*. Camphor fed at 119 ppm protected weight gains and protected against *E. tenella* and *E. acervulina* [95].

The essential oil of absinthe wormwood (*Artemisia absinthium*), containing 27.40% camphor, showed activity against promastigote (MIC 0.0097 μL/mL) and axenic amastigote forms (EC_50_ 0.24 nL/mL) of both *Leishmania aethiopica* and *L. donovani*. It also showed a weak haemolytic effect with a slightly decreased selectivity index (SI = 0.8) against the THP-1 cell line. This study demonstrated the potential use of *Artemisia absinthium* oil as source of an innovative agent for the treatment of leishmaniasis [96].

### 5.9. Allelopathic Activity

Allelopathy is the interaction of one plant with another through the release of biochemical compounds into the environment and can be indirect, direct, harmful or beneficial. It can occur through several mechanisms including decay with or without micro-organisms, excretion, exudation, volatilisation and leaching. Allellochemicals can be present in any plant part as well as in the surrounding soil and are mostly secondary metabolites and include alkaloids, phenyl propanes, naphthaquinones, steroids and terpenoids amongst others. Allelopathic activity can lead to suppression of growth of one plant by another [97]. In a significant study by Schenk [97], the allelopathic influence of the camphor laurel tree (*C. camphora*) was investigated on the seedling growth of fifty-two plant species and twenty-seven soil algal populations. The leaves had a direct allelopathic effect by significantly delaying germination and causing a reduction of radicle and shoot length, leaf area and leaf number, specifically in species associated with camphor laurel (*C. camphora*) communities. In addition, many of the soil algal species disappeared from the soil or exhibited reduced vigour, therefore the allelopathic effect may also be indirect as soil algae are necessary for soil wettability, moisture retention and seed germination enhancement. The persistence and dominance of camphor laurel trees (*C. camphora*) may also be enhanced as the allelopathic activity reduced the competitiveness of surrounding vegetations [97]. In another study, the chemical release from leaf powder of the camphor laurel tree (*C. camphora*) was studied by monitoring soil and air concentrations of (+)-camphor. The growth of the receiver plant—rice seedlings—was inhibited when planted in soil which contained the leaf powder. (+)-Camphor was detected in this soil as well as the soil water and it was therefore determined to be the main phytotoxic allelochemical responsible for the growth suppression [98]. The allelopathic activity of camphor and other monoterpenes were studied by determining the antigerminative ability in radish (*Raphanus sativus*) and garden cress (*Lepidium sativum*) seeds 120 h after sowing. The radish (*R. sativus*) seeds were found to be more sensitive than the garden cress (*L. sativum*) seeds and at 10^−3^ M, camphor significantly inhibited the germination of radish (*Raphanus sativus*) seedlings by 44% and affected the radicle growth of garden cress (*Lepidium sativum*) seeds. From this study, the authors concluded that monoterpenes such as camphor, which exhibits phytotoxic activity, are therefore potential bio-herbicides which could be developed into natural pesticides [99].

## 6. Toxicity of Camphor

The toxicity of camphor has been well-documented. The ingestion of 3.5 g of camphor can cause death, whilst 2.0 g causes toxic effects in adults leading to congestion of the gastrointestinal tract, kidney and brain; the immediate collapse of an infant has been reported after the application of a small dose to the nostrils [100]. In humans, the characteristic symptoms of camphor poisoning after ingestion are nausea, vomiting, headache, dizziness, muscular excitability causing tremor and twitching, convulsions and delirium depending on the dosage. In a severe overdose, status epilepticus persisting for several hours occurs, ultimately causing coma and death by asphyxia or exhaustion [101,102,103,104]. Camphor inhalation may cause irritation of the mucous membranes above 2 ppm and respiratory depression and apnoea may occur. Camphor can also cause skin and eye irritation on contact. Inhalation and skin exposure may result in acute poisoning depending on the dose with symptoms described above. After ingestion, rapid onset of vomiting improves the prognosis but simultaneous intake of alcohol, or oily solutions, enhance the absorption of camphor and therefore the toxic effect. Camphor poisoning is treated symptomatically as no antidote is known [102].

In response to several toxicity reports, the United States Food and Drug Administration evaluated the efficacy and toxicity of camphor-containing products. A limit of 11% in consumer products was set in 1983 and products labeled as camphorated oil, camphor oil, camphor liniment and camphorated liniment were banned completely [105]. However in most countries, especially in developing countries, medicaments containing camphor as high as 20% are easily available. According to 2001 data from the American Association of Poison Control Centers Toxic Exposure Surveillance System (TESS), 8,505 exposures to camphor products were reported, many of which resulted in mild symptoms with 89 moderate to severe outcomes but no fatalities [106].

The majority of drugs administered to humans and animals are eliminated by a combination of hepatic metabolism and renal excretion [107]. In the human body, camphor is oxygenated to alcohol campherol and then conjugated with glucuronic acid in the liver to become soluble in water before being excreted in the urine. Following oral ingestion, high concentrations of camphor have been detected in the foetal brain, liver, kidney, blood, as well as in amniotic fluid [108]. The exact mechanism of camphor-induced toxicity has not been completely elucidated, but a study by Park *et al*. [109] indicated camphor specifically inhibits the nicotinic acethylcholine receptors (nAChRs), thereby causing inhibition of catecholamine secretion. This inhibition may be one cause of the toxicity as nAChRs are known to play a major role at neuromuscular junctions [109]. Camphor ingestion may lead to abortion as it crosses the placenta and foetuses lack the enzymes to hydroxylate and conjugate with glucuronic acid [110]. No teratogenicity was noted during the foetal period of organogenesis after oral (+)-camphor administration to pregnant rats and rabbits at doses up to 1,000 mg/kg bodyweight/day and 681 mg/kg bodyweight/day respectively. However, the high dose of 1,000 mg/kg bodyweight/day caused toxic symptoms including clonic convulsions, pilo-erection and reduced motility in rats, but no retardations or malformations were observed. In the rabbit model a high dose of 681 mg/kg bodyweight/day resulted in reduced body weight gain and food consumption, but no retardations or malformations were observed [111].

A study investigating the toxicological effects of camphor on the rabbit (*Oryctolagus cuniculus*) kidney involved the oral administration of different concentrations of camphor solution for a period of ten days, which resulted in mild oedema, glomerulonephritis, glomerular lobulations, tubular necrosis and congestion of the blood cells. Histologically, camphor administration distorted and disrupted the cytoarchitecture of the kidney [112]. Central serous chorioretinopathy (CSCR) of the right eye was reported in a 50-year old female of Chinese origin who had used Chinese herbal medicinal patches containing camphor as the main ingredient for more than 20 years [113].

As mentioned previously, camphor is a major component of many aromatic plant species. Millet *et al.* [114] investigated the toxicity of some commercial essential oils including sage (*Salvia officinalis*), hyssop (*Hyssopus officinalis*), thuja (*Thuja occidentalis*) and cedar (*Juniperus* and *Cupressus* spp.). For sage (*Salvia officinalis*) oil, 3.2 g/kg of sage oil caused tonic-clonic convulsions in unanaesthetised rats resulting in death. It was determined that the toxicity of sage (*Salvia officinalis*) oil appeared to be related to the presence of camphor [114].

## 7. Conclusions

Camphor is a multipurpose molecule with a most diverse range of applications, ranging from being used to treat medical conditions in humans to being used as a natural poison to kill insects, which seems divergent. In fact, the toxicity of camphor in humans remains a cause for concern as many cases of accidental poisoning, with serious symptoms, have occurred. However not only pure camphor should be considered, it is important to remember many products, plants and essential oils contain camphor. The overwhelmingly distinct aroma of camphor has led to its wide use in ointments and inhalants, particularly as an adjunct to treat the common cold. Scientifically, numerous biological activities have been attributed to camphor including antibacterial, antifungal, antimutagenic, antitussive and insecticidal properties, but it is important to note that bioactivity was determined in many cases using an essential oil rich in camphor and not pure camphor. Due to the high percentage of camphor, these activities may be incorrectly attributed to camphor, whilst synergism seems much more likely as was shown in the example of 1,8-cineole and (−)-camphor. Other studies showed pure camphor did not possess the same activity as the neat essential oil. Clearly, if these properties are to be confirmed, further research on camphor alone needs to follow up on these essential oil studies. In addition to its many medicinal uses, camphor is a useful molecule in chemical reactions where it is used extensively as a catalyst and has served as a chiral starting material and auxiliary. It is evident from this review that camphor is a most versatile molecule with a multitude of applications.

## References

[1] Keller H. (2009). The World I Live in.

[2] Vernet-Maury E., Alaoui-Ismaïli O., Dittmar A., Delhomme G., Chanel J. (1999). Basic emotions induced by odorants: A new approach based on autonomic pattern results. J. Auton. Nerv. Syst..

[3] Van Wyk B.E., van Oudtshoorn B., Gericke N. (2009). Medicinal plants of South Africa.

[4] United States Department of Agriculture (USDA), Natural Resources Conservation Service *Cinnamomum camphora* (L.) J. Presl (camphor tree). http://plants.usda.gov/java/profile?symbol=CICA&photoID=cica_002_ahp.tif/.

[5] Hattori A. (2001). Camphor in the Edo era fireworks. Yakushiqaku Zasshi.

[6] Donkin R.A. (1999). Dragon’s brain Perfume: An Historical Geography of Camphor.

[7] Kumar M., Ando Y. (2003). Single-wall and multi-wall carbon nanotubes from camphor-a botanical hydrocarbon. Diamond Relat. Mater..

[8] Gomes-Carneiro M.R., Felzenszwalb I., Paumgartten F.J. (1998). Mutagenicity testing (+/−)-camphor, 1,8-cineole, citral, citronellal, (−)-menthol and terpineol with the *Salmonella*/microsome assay. Mutat. Res..

[9] Liu W., Ager D.J. (2005). Terpenes: The expansion of chiral pool. Handbook of Chiral Chemicals.

[10] Juteau F., Masotti V., Bessière J.M., Dherbomez M., Viano J. (2002). Antibacterial and antioxidant activities of *Artemisia annua* essential oil. Fitoterapia.

[11] Tirillini B., Velasquez E.R., Pellegrino R. (1996). Chemical composition and antimicrobial activity of essential oil of *Piper angustifolium*. Planta Med..

[12] Kamdem D.P., Gage D.A. (1995). Chemical composition of essential oil from the root bark of *Sassafras albidum*. Planta Med..

[13] Viljoen A., van Vuuren S., Ernst E., Klepser M., Demirci B., Baser H., van Wyk B. (2003). *Osmitopsis astericoides* (Asteraceae)—The antimicrobial activity and essential oil composition of a Cape-Dutch remedy. J. Ethnopharmacol..

[14] Hammerschmidt F.J., Clark A.M., Soliman F.M., El-Kashoury E.S., Abd El-Kawy M.M., El-Fishawy A.M. (1993). Chemical composition and antimicrobial activity of essential oils of *Jasonia candicans* and *J. montana*. Planta Med..

[15] Philpott N.W. (1929). Intramuscular Injections of camphor in the treatment of engorgement of the breasts. CMAC.

[16] Liebelt E.L., Shannon M.W. (1993). Small doses, big problems: A selected review of highly toxic common medications. Pediatr. Emerg. Care.

[17] Mann J.C., Hobbs J.B., Banthorpe D.V., Harborne J.B. (1994). Natural Products: Their Chemistry and Biological Significance.

[18] Bauer K., Garbe D., Surburg H. (1997). Common Fragrance and Flavor Materials.

[19] Nandi N. (2005). Study of chiral recognition of model peptides and odorants: Carvone and camphor. Curr. Sci..

[20] Rebound Health Camphor. reboundhealth.com/cms/images/pdf/Textbooks/camphor%20id%2015853.pdf.

[21] Lopes-Lutz D., Alviano D.S., Alviano C.S., Kolodziejczyk P.P. (2008). Screening of chemical composition, antimicrobial and antioxidant activities of *Artemisia* essential oils. Phytochemistry.

[22] Viljoen A.M., Njenga E.W., van Vuuren S.F., Bicchi C., Rubiolo P., Sgorbini B. (2006). Essential oil composition and *in vitro* biological activities of seven Namibian species of *Eriocephalus* L. (Asteraceae). JEOR.

[23] Damjanoviæ-Vratnica B., Dakov T. , Šukoviæ D., Damjanoviæ J. (2008). Chemical composition and antimicrobial activity of essential oil of wildgrowing *Salvia officinalis* L. from Montenegro. JEOBP.

[24] Kelen M., Tepe B. (2008). Chemical composition, antioxidant and antimicrobial properties of the essential oils of three *Salvia* species from Turkish flora. Bioresour. Technol..

[25] Croteau R., Hooper C.L., Felton M. (1979). Biosynthesis of monoterpenes. Partial purification and characterization of a bicyclic monoterpenol dehydrogenase from sage (*Salvia officinalis*). Arch. Biochem. Biophys..

[26] Croteau R., Karp F. (1979). Biosynthesis of monoterpenes: Preliminary characeterisation of bornyl pyrophosphate synthetase from sage (*Salvia officinalis*) and demonstration that geranyl pyrophosphate is the preferred substrate for cyclization. Arch. Biochem. Biophys..

[27] Croteau  R., Karp F. (1979). Biosythesis of monoterpenes: Hydrolysis of bornyl pyrophosphate, an essential step in camphor biosynthesis, and hydrolysis of geranyl pyrophosphate, the acyclic precursor of camphor, by enzymes from sage (*Salvia officinalis*). Arch. Biochem. Biophys..

[28] Croteau R., Felton M., Karp F., Kjonaas R. (1981). Relationship of camphor biosynthesis to leaf development in sage (*Salvia officinalis*). Plant Phys..

[29] Jiang Y., Guo P., Liu G. (1990). Asymmetric Synthesis X: The high enantioselective synthesis of (R)-α-substituted benzylic amines via the modified (+)-camphor derivative as chiral synthon. Synth. Commun..

[30] Stevens R.V., Gaeta C.A. (1977). Camphorae: Chiral intermediates for the total synthesis of steroids. J. Am. Chem. Soc..

[31] Li T., Du P., Sun Y., Lin L., Liu Y. (2011). Synthesis of vitcamphor derivatives of camphor and its preliminary anti-inflammatory activity. HHBE.

[32] Magiatis P., Skaltsounis A.L., Chinou I., Haroutounian S.A. (2002). Chemical composition and *in vitro* antimicrobial activity of the essential oils of three Greek *Achillea* species. Z. Naturforsch. C.

[33] De Heluani C.S., de Lampasona M.P., Vega M.I., Catalan C.A.N. (2005). Antimicrobial activity and chemical composition of the leaf and root oils from *Croton hieronymi* Griseb. JEOR.

[34] Zhu S., Yang Y., Yu H., Ying Y., Zou G. (2005). Chemical composition and antimicrobial activity of the essential oils of *Chrysanthemum indicum*. J. Ethnopharmacol..

[35] Kotan R., Kordali S., Cakir A., Kesdek M., Kaya Y., Kilic H. (2008). Antimicrobial and insecticidal activities of essential oil isolated from Turkish *Salvia hydrangea* DC: Ex Benth. Biochem. Syst. Ecol..

[36] Setzer W.N., Vogler B., Schmidt J.M., Leahy J.G., Rives R. (2004). Antimicrobial activity of *Artemisia douglasiana* leaf essential oil. Fitoterapia.

[37] Soković M., van Griensven L.J.L.D. (2006). Antimicrobial activity of essential oils and their components against the three major pathogens of the cultivated button mushroom, *Agaricus bisporus*. Eur. J. Plant Pathol..

[38] Sivropoulou A., Nikolaou C., Papanikolaou E., Kokkini S., Lanaras T., Arsenakis M. (1997). Antimicrobial, cytotoxic and antiviral activities of *Salvia fruticosa* essential oil. J. Agric. Food Chem..

[39] Santoyo S., Cavero S., Jaime L., Ibañez E., Señoráns F.J., Reglero G. (2005). Chemical composition and antimicrobial activity of *Rosmarinus officinalis* L. essential oil obtained via supercritical fluid extraction. J. Food Prot..

[40] Sökmen A., Vardar-Ünlü G., Polissiou M., Daferera D., Sökmen M., Dönmez E. (2003). Antimicrobial activity of essential oil and methanol extracts of *Achillea sintenisii* Hub. Mor. (Asteraceae). Phytother. Res..

[41] Mevy J.P., Bessiere J.M., Dherbomez M., Millogo J., Viano. J. (2007). Chemical composition and some biological activities of the volatile oils of a chemotype of *Lippia chevalieri* Moldenke. Food Chem..

[42] Ouattara B., Simard R.E., Holley R.A., Piette G.J.P., Bégin A. (1997). Antibacterial activity of selected fatty acids and essential oils against six meat spoilage organisms. Int. J. Food Microbiol..

[43] Tabanca N., Demirci B., Başer K.H.C., Aytac Z., Ekici M., Khan S.I., Jacob M.R., Wedge D.E. (2006). Chemical composition and antifungal activity of *Salvia macrochlamys* and *Salvia recognita* essential oils. J. Agric. Food Chem..

[44] De Logu A., Loy G., Pellerano M.L., Bonsiqnore L., Schivo M.L. (2000). Inactivation of HSV-1 and HSV-2 and prevention of cell-to-cell virus spread by *Santolina insularis* essential oil. Antiviral Res..

[45] Burrow A., Eccles R., Jones A.S. (1983). The effects of camphor, eucalyptus and menthol vapour on nasal resistance to airflow and nasal sensation. Acta Otolaryngol..

[46] Laude E.A., Morice A.H., Grattan T.J. (1994). The antitussive effects of menthol, camphor and cineole in conscious guinea-pigs. Pulm. Pharmacol..

[47] McKemy D.D., Neuhausser W.M., Julius D. (2002). Identification of a cold receptor reveals a general role for TRP channels in thermosensation. Nature.

[48] McKemy D.D. (2005). How cold is it? TRPM8 and TRPA1 in the molecular logic of cold sensation. Mol. Pain.

[49] Kumar N., Nepali K., Sapra S., Bijjem K.R.V., Kumar R., Suri O.P., Dhar K.L. (2012). Effect of nitrogen insertion on the antitussive properties of menthol and camphor. Med. Chem. Res..

[50] Green B.G. (1990). Sensory characteristics of camphor. J. Invest. Dermatol..

[51] Xu H., Blair N.T., Clapham D.E. (2005). Camphor activates and strongly desensitizes the transient receptor potential vanilloid subtype 1 channel in a vanilloid-independent mechanism. J. Neurosci..

[52] Adams J.D. (2012). The use of California sagebrush (*Artemisia californica*) liniment to control pain. Pharmaceuticals.

[53] Ghanta V.K., Hiramoto N.S., Solvason H.B., Tyring S.K., Spector N.H., Hiramoto R.N. (1987). Conditioned enhancement of natural killer cell activity, but not interferon, with camphor or saccharin-LiCl conditioned stimulus. J. Neurosci. Res..

[54] Banerjee S., Welsch C.W., Rao A.R. (1995). Modulatory influence of camphor on the activities of hepatic carcinogen metabolizing enzymes and the levels of hepatic and extrahepatic reduced glutathione in mice. Cancer Lett..

[55] Goel H.C., Roa A.R. (1988). Radiosensitizing effect of camphor on transplantable mammary adenocarcinoma in mice. Cancer Lett..

[56] Goel H.C., Singh S., Singh S.P. (1989). Radiomodifying influence of camphor on sister-chromatid exchange induction in mouse bone marrow. Mutat. Res..

[57] Kanematsu N., Shibata K.I. (1990). Investigation of DNA reactivity of endodontic agents by rec-assay. Gifu Shika Gakkai Zasshi.

[58] Simić D., Vuković-Gacić B., Knezević-Vukcević J. (1998). Detection of natural bioantimutagens and their mechanisms of action with bacterial assay-system. Mutat. Res..

[59] Vuković-Gacić B., Nikcević1 S., Berić-Bjedova T., Knezević-Vukcević J., Simić D. (2006). Antimutagenic effect of essential oil of sage (*Salvia officinalis* L.) and its monoterpenes against UV-induced mutations in *Escherichia coli* and *Saccharomyces cerevisiae*. Food Chem. Toxicol..

[60] Nikolić B., Mitić-Ćulafić D., Vuković-Gacić B., Knezević-Vukcević J. (2011). Modulation of genotoxicity and DNA repair by plant monoterpenes camphor, eucalyptol and thujone in *Escherichia coli* and mammalian cells. Food Chem. Toxicol..

[61] De-Oliveira A.C., Ribeiro-Pintob L.F., Paumgartten F.J.R. (1997). *In vitro* inhibition of CYP2B1 monooxygenase by beta-myrcene and other monoterpenoid compounds. Toxicol. Lett..

[62] (1998). Methyl bromide technical options committee (MBTOC): Assessment of alternatives to methyl bromide. Nairobi, Kenya, United Nations Environment Programme, Ozone Secretariat. http://ozone.unep.org/Assessment_Panels/TEAP/Reports/MBTOC/MBTOC-Assesment-Report-2010.pdf.

[63] Li Y.S., Zou H.Y. (2001). Insecticidal activity of extracts from. *Eupatorium adenophorum* against four stored grain insects. Entomol. Knowl..

[64] Silva W.J., Dória G.A.A., Maia R.T., Nunes R.S., Carvalho G.A., Blank A.F., Alves P.B., Marçal R.M., Cavalcanti S.C.H. (2008). Effects of essential oils on *Aedes aegypti* larvae: Alternatives to environmentally safe insecticides. Bioresour. Technol..

[65] Whittaker R.H., Sondheimer E., Simeone J.B. (1970). The biochemical ecology of higher plants. Chemical Ecology.

[66] Brattsten L.B., Hedin P.A. (1983). Cytochrome P-450 involvement in the interactions between plant terpenes and insect herbivores. Plant Resistance to Insects.

[67] Obeng-Ofori D., Reichmuth C.H., Bekele A.J., Hassanali A. (1998). Toxicity and protectant potential of camphor, a major component of essential oil of *Ocimum kilimandscharicum*, against four stored product beetles. Int. J. Pest Manag..

[68] Bekele J., Hassanali A. (2001). Blend effects in the toxicity of the essential oil constituents of *Ocimum kilimandscharicum* and *Ocimum kenyense* (Labiateae) on two post-harvest insect pests. Phytochemistry.

[69] Rozman V., Kalinovic I., Korunic Z. (2006). Toxicity of naturally occurring compounds of Lamiaceae and Lauraceae to three stored-product insects. J. Stored Prod. Res..

[70] Liska A., Rozman V., Kalinovic I., Ivecic M., Balicevic R. (2010). Contact and fumigant activity of 1,8-cineole, eugenol and camphor against *Tribolium castaneum* (Herbst).

[71] Qiantai L., Yongcheng S., Zuxun J., Quan L., Yongsheng L., Xianchang T., Lianghua G. (1998). Studies on effect of several plant materials against stored grain insects. Proceedings of the Seventh International Conference on Stored-Product Protection.

[72] Abivardi C., Zareh N. (1977). Effect of camphor on embryonic and postembryonic development of *Callosobruchus chinensis*. J. Econ. Entomol..

[73] Riddick E.W., Aldrich J.R., de Milo A., Davis J.C. (2000). Potential for modifying the behavior of the multi-colored Asian lady beetle (Coleoptera: Coccinellidae) with plant-derived natural products. Ann. Entomol. Soc. Am..

[74] Ojimelukwe P.C., Adler C. (2000). Toxicity and repellent effects of eugenol, thymol, linalool, menthol and other pure compounds on *Dinoderus bifloveatus* (Coleoptera: Bostrichidae). J. Sustain. Agric. Environ..

[75] Prates H.T., Leite R.C., Craveiro A.A., Oliveira A.B. (1998). Identification of some chemical components of the essential oil from molasses grass (*Melinis minutiflora* Beauv.) and their activity against cattle-tick (*Boophilus microplus*). J. Braz. Chem. Soc..

[76] Arlian L.G. (2002). Arthropod allergens and human health. Annu. Rev. Entomol..

[77] Jung W.C., Jang Y.S., Hieu T.T., Lee C.K., Ahn Y.J. (2007). Toxicity of *Myristica fragrans* seed compounds against *B. germanica* (Dictyoptera: Blattellidae). J. Med. Entomol..

[78] Pohlit A.M., Lopes N.P., Gama R.A., Tadei W.P., de Andrade Neto V.F. (2011). Patent literature on mosquito repellent inventions which contain plant essential oils—A review. Planta Med..

[79] Briassoulis G., Narlioglou M., Hatzis T. (2001). Toxic encephalopathy associated with use of DEET insect repellents: A case analysis of its toxicity in children. Hum. Exp. Toxicol..

[80] Gillij Y.G., Gleiser R.M., Zygadlo J.A. (2008). Mosquito repellent activity of essential oils of aromatic plants growing in Argentina. Bioresour. Technol..

[81] Ansari M.A., Razdan R.K. (1995). Relative efficacy of various oils in repelling mosquitoes. Indian J. Malariol..

[82] Seyoum A., Palsson K., Kung’a S., Kabiru E.W., Lwande W., Killeen G.F., Hassanali A., Knols B.G. (2002). Traditional use of mosquito-repellent plants in western Kenya and their evaluation in semi-field experimental huts against *Anopheles gambiae*: Ethnobotanical studies and application by thermal expulsion and direct burning. Trans. R. Soc. Trop. Med. Hyg..

[83] Seyoum A., Killeen G.F., Kabiru E.W., Knolls B.G.J., Hassanali A. (2003). Field efficacy of thermally expelled or live potted repellent plants against African malaria vectors in western Kenya. Trop. Med. Int. Health.

[84] Osborne O.T. (1928). Camphor and strychnine as cardiac stimulants. JAMA.

[85] Belz G.G., Breithaupt-Grögler K., Butzer R., Herrmann V., Malerczyk C., Mang C., Roll S., Rietbrock N. (2000). Klinische pharmakologie von D-Campher. Phytopharmaka VI.

[86] Belz G.G., Loew D. (2003). Dose-response related efficacy in orthostatic hypotension of a fixed combination of D-camphor and an extract from fresh *Crataegus* berries and the contribution of the single components. Phytomedicine.

[87] Williams A.C., Barry B.W. (1991). Terpenes and the lipid-protein-partitioning theory of skin penetration enhancement. Pharmaceut. Res..

[88] Yano T., Kanetake T., Saita M., Noda K. (1991). Effects of l-menthol and dl-camphor on the penetration and hydrolysis of methyl salicylate in hairless mouse skin. J. Pharmacobiodyn.

[89] Fang J.Y., Tsai T.H., Lin Y.Y., Wong W.W., Wang M.N., Huang J.F. (2007). Transdermal delivery of tea catechins and theophylline enhanced by terpenes: A mechanistic study. Biol. Pharm. Bull..

[90] Ramesh G., Vamshi V.Y., Kishan V., Madhusudan R.Y. (2007). Studies on the influence of penetration enhancers on *in vitro* permeation of carvedilol across rat abdominal skin. Curr. Trends Biotechnol. Pharm..

[91] Jain R., Aqil M., Ahad A., Ali A., Khar R.K. (2008). Basil oil is a promising skin penetration enhancer for transdermal delivery of labetolol hydrochloride. Drug Develop. Ind. Pharm..

[92] Liu H., Zhou Y., Sun Y., Sheng X., Zhang J., Zhang Z., Ding J. (2011). Effect of menthol and camphor on permeation of compound diphenhydramine cream *in vitro*. Cent. South Pharm..

[93] Jamshidzadeh A., Sajedianfard J., Nekooeian A.A., Tavakoli F., Omrani G.H. (2006). Effects of Camphor on Sexual Behaviors in Male Rats. IJPS.

[94] Nikravesh M.R., Jalali M. (2004). The effect of camphor on the male mice reproductive system. Urol. J..

[95] Allen P.C., Lydon J., Danforth H.D. (1997). Effects of components of *Artemisia annua* on coccidia infections in chickens. Poult Sci..

[96] Tariku Y., Hymete A., Hailu A., Rohloff J. (2011). *In vitro* evaluation of antileishmanial activity and toxicity of essential oils of *Artemisia absinthium* and *Echinops kebericho*. Chem. Biodivers..

[97] Schenk J.R. (2009). Phytochemistry, allelopathy and the capability attributes of camphor laurel (*Cinnamomum camphora* (L.) Ness & Eberm.). Ph.D. Thesis.

[98] Okamoto Y., Yamahi K., Kobayashi K. (2011). Allelopathic activity of camphor released from camphor tree (*Cinnamomum camphora*). Allelopathy J..

[99] De Martino L., Mancini E., de Almeida L.F.R., de Feo V. (2010). The antigerminative activity of twenty-seven monoterpenes. Molecules.

[100] Arena J.M. (1979). Poisoning: Toxicology, Symptoms, Treatments.

[101] Rabl W., Katzgraber F., Steinlechner M. (1997). Camphor ingestion for abortion (case report). Forensic Sci. Int..

[102] International program on chemical safety (IPCS) INCHEM Camphor. http://www.inchem.org/documents/pims/pharm/camphor.htm/.

[103] Love J.N., Sammon M., Smereck J. (2003). Are one or two dangerous? Camphor exposure in toddlers. J. Emerg. Med..

[104] Phelan W.J. (1976). Camphor poisoning: Over-the-counter dangers. Pediatrics.

[105] Theis J.G., Koren G. (1995). Camphorated oil: Still endangering the lives of Canadian children. CMAJ.

[106] Manoguerra A.S., Erdman A.R., Wax P.M., Nelson L.S., Caravati E.M., Cobaugh D.J., Chyka P.A., Olson K.R., Booze L.L., Woolf A.D. (2006). Camphor Poisoning: An evidence-based practice guideline for out-of-hospital management. Clin. Toxicol..

[107] Katzung G.B. (1998). Basic and Clinical Pharmacology.

[108] Gosselin R.E., Smith R.P., Hodge H.C. (1984). Clinical Toxicology of Commercial Products.

[109] Park T., Seo H., Kang B., Kim K. (2001). Noncompetitive inhibition by camphor of nicotinic acetylcholine receptors. Biochem. Pharmacol..

[110] Riggs J., Hamilton R., Homel S., McCabe J. (1965). Camphorated oil intoxication in pregnancy; report of a case. Obstet. Gynecol..

[111] Leuschner J. (1997). Reproductive toxicity studies of *d*-camphor in rats and rabbits. Arzneim. Forsch..

[112] Enaibe B., Eweka A., Adjene J. (2008). Toxicological effects of camphor administration on the histology of the kidney of the rabbit (*Oryctolagus cuniculus*). Internet J. Toxicol..

[113] Kahook M.Y., Thomas S.A., Ciardella A.P. (2007). Central serous chorioretinopathy associated with chronic dermal camphor application. Internet J. Ophthalmol. Vis. Sci..

[114] Millet Y., Jouglard J., Steinmetz M.D., Tognetti P., Joanny P., Arditti J. (1981). Toxicity of some essential plant oils. Clinical and experimental study. Clin. Toxicol..

